# Development of Posterior Lenticonus Following the Diagnosis of Isolated Anterior Lenticonus in Alport Syndrome

**DOI:** 10.7759/cureus.12970

**Published:** 2021-01-28

**Authors:** Lubna M Halawani, Mohammed F Abdulaal, Hammam A Alotaibi, Anoud F Alsaati, Turki A Bin Dakhil

**Affiliations:** 1 Department of Neurology, King Fahad Medical City, Riyadh, SAU; 2 Department of Ophthalmology, Ohud Hospital, Medina, SAU; 3 Research Center, Prince Sultan Military Medical City, Riyadh, SAU; 4 Department of Ophthalmology, Prince Sultan Military Medical City, Riyadh, SAU

**Keywords:** lenticonus, alport, alport syndrome, posterior lenticonus

## Abstract

Alport syndrome is a genetic disorder that manifests as renal disease, hearing loss and ocular dysfunction. Lenticonus is one such ocular condition, in which the lens takes on an abnormal cone shape, with a protrusion either at the front or back of the lens. Both sides of the lens are rarely affected at the same time in the general patient population. Although anterior lenticonus is the type that is often reported in Alport syndrome, it is rare for such patients to have both anterior and posterior lenticonus. Here, the case of a 32-year-old male with Alport syndrome is described. The patient was diagnosed with a progressive posterior lenticonus, having been diagnosed eight years earlier with isolated anterior lenticonus. Examination of the eye revealed the typical indications of lenticonus with flecked retinopathy. The patient had co-presenting astigmatism and a refractive error, which could not be corrected by wearing contact lenses or spectacles. It is critical that such cases are anticipated and identified prior to performing surgery, so that an appropriate approach can be taken, thereby minimizing surgical complications.

## Introduction

Lenticonus is an uncommon morphological abnormality in which the lens capsule thins and bulges, adopting a conical shape. This change can affect either the anterior or the posterior surfaces. Both surfaces of the lens can be affected at the same time but this is very rare. The diameter of the conical protrusion can be between two to seven millimeters. Lenticonus can be confused with the similar condition, lentiglobus, in which the lens has a spherical protuberance [1]. Patients with lenticonus typically co-present with astigmatism and myopia. In addition to anterior lenticonus, Alport syndrome can manifest as cataracts, corneal arcus juvenilis, posterior polymorphous dystrophy, and flecked retinopathy. On the other hand, posterior lenticonus is a congenital condition and it is not linked to systemic disease. However, reports in the literature suggest it could be associated with Lowe’s syndrome [2]. This case report describes a case in which the patient diagnosed with a progressive posterior lenticonus, having been diagnosed eight years earlier with isolated anterior lenticonus. It is exceptionally rare to find reports of simultaneous presentations of anterior and posterior lenticonus at diagnosis [3,4]. Indeed, only one such other case has been reported. In that instance, progressive posterior lenticonus was diagnosed 18 months after the initial anterior lenticonus diagnosis [5].

## Case presentation

**Figure 1:**
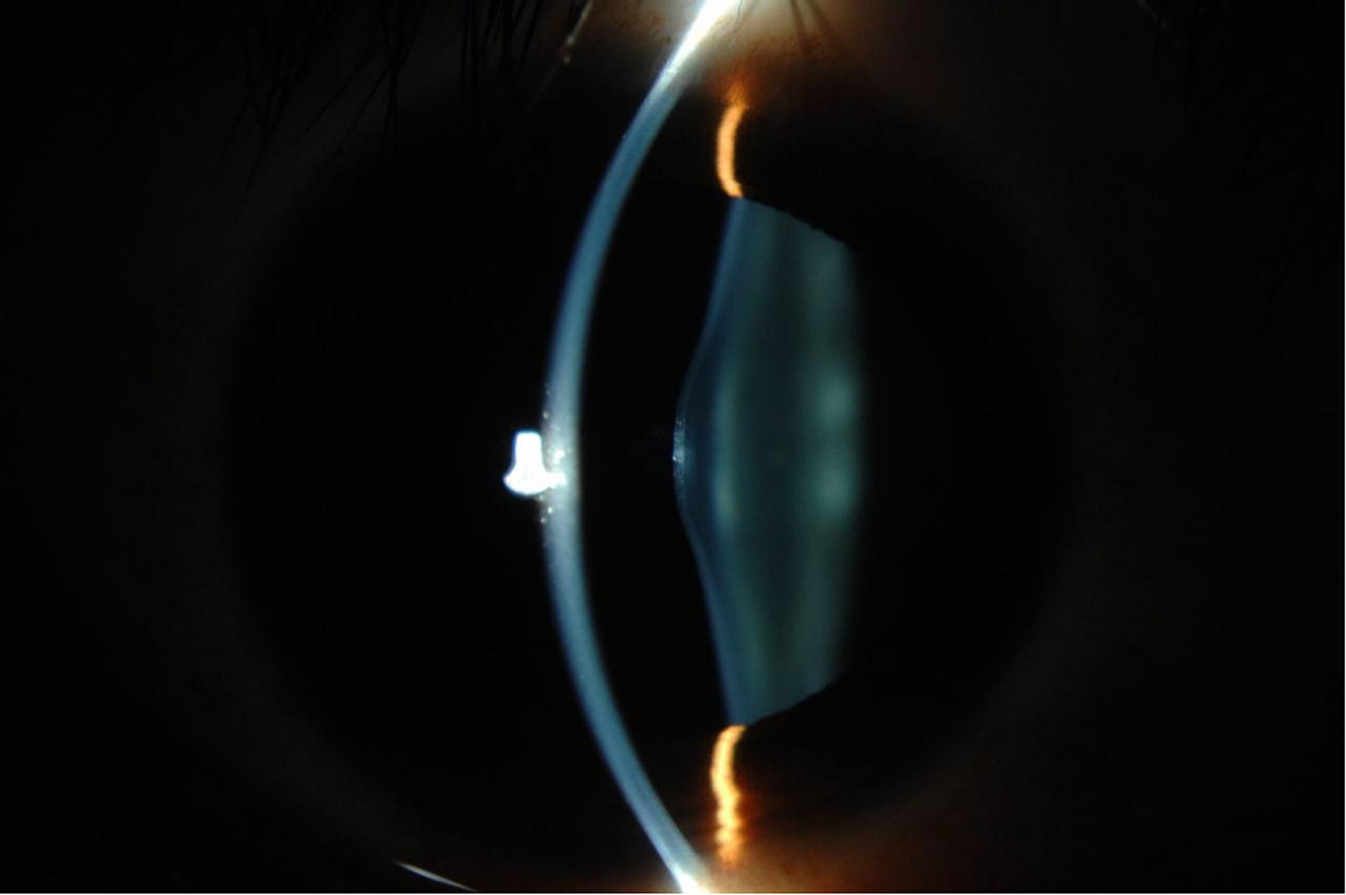
Slit-lamp photo showing anterior lenticonus as observed in both eyes of the patient

A 32-year-old male, Alport syndrome patient came to the ophthalmology clinic with a complaint of progressive loss of vision in both eyes, since childhood. Elsewhere, he had been diagnosed with bilateral isolated anterior lenticonus (Figure 1). Following a lens aspiration operation, he had received an in-the-bag intraocular lens (IOL) implantation in his right eye. At the same time, he had been referred to the nephrology department for a renal transplant and to an otologist for his hearing impairment. These referrals interfered with his ophthalmological follow-up. It was eight years later that he came for a follow-up at our tertiary care hospital.

**Figure 2:**
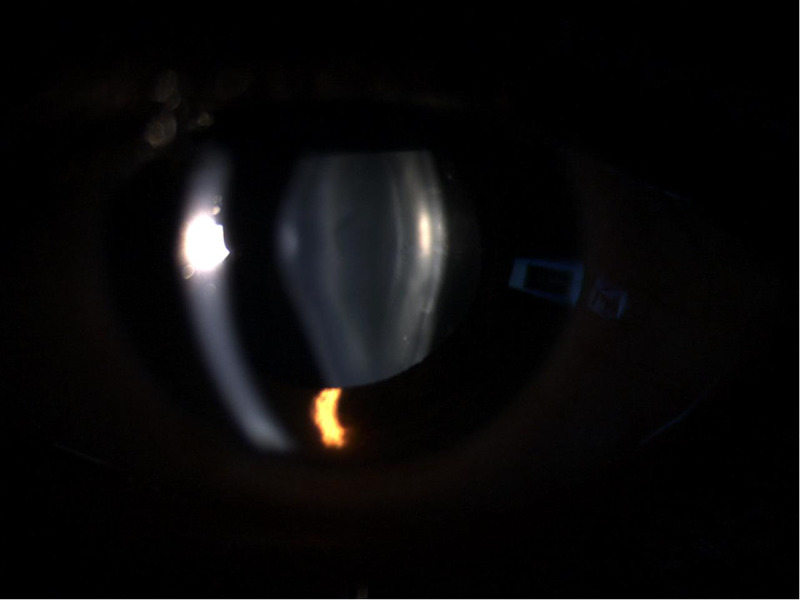
Slit-lamp photo of the left eye showing development of posterior lenticonus

The follow-up examination showed that in the intervening years between visits to the ophthalmology clinic, a posterior lenticonus had developed in his left eye (Figure 2).

**Figure 3:**
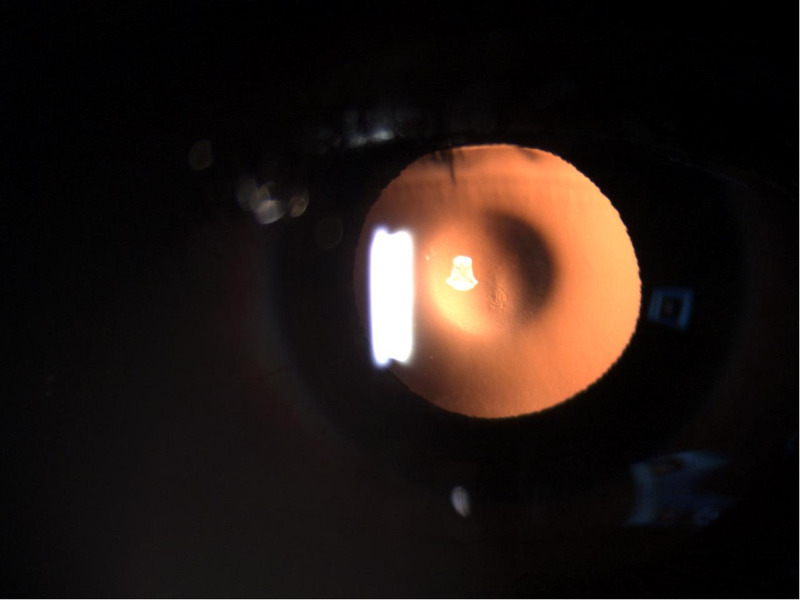
Slit-lamp photo showing the classic oil-droplet appearance associated with fleck retinopathy

Ocular examination of the right eye revealed the patient’s best-corrected distance visual acuity was 20/25, but his left eye was 20/100. In the right eye, the manifest refraction was +0.75-1.25X005, and in the left eye it was plano -2.00X180. The patient’s tomographic astigmatism was -1.64 at 007 in the right eye and -1.49 at 002 in the left eye. Axial length in the right eye was 22.76 mm, and in the left eye, 23.02 mm. Qualitative and quantitative assessment of specular microscopy results were normal for his age. The intraocular pressure in both eyes was normal. Slit-lamp examination of the right eye showed the cornea to be clear, intraocular lens in in-the-bag and evidence of fleck retinopathy. The same examination of the left eye also revealed a clear cornea but a finding of anterior and posterior lenticonus, measuring three and four millimeters, respectively. The classic oil-droplet appearance that is associated with fleck retinopathy was also observed (Figure 3).

## Discussion

Alport syndrome is an X-linked disorder that affects at least one in 10,000 people. It is associated with three characteristic pathologies: progressive nephritis with hematuria, ocular abnormalities and sensorineural hearing loss [6]. Because it is a heritable condition, correct diagnosis is vital to identify other members of the family who may have inherited the disease mutations. Early diagnosis results in a better prognosis as prompt treatment can delay the onset of end-stage renal failure [7]. Alport syndrome ocular pathogenesis, such as lenticonus, arises from the loss of type IV collagen α3α4α5 network in the basement membranes of different structures of the eyes including the lens [8]. The conical protrusion of the lens in lenticonus occurs at the thinnest and weakest anterior part of the capsule [9]. Anterior lenticonus with X-linked disease is observed in 50% of men, but not reported in women. The condition is linked to perimacular retinopathy and early onset renal failure. In contrast, lenticonus due to inheritance of autosomal recessive genes does occur in both males and females; therefore, females with Alport syndrome and lenticonus most likely have inherited the recessive gene [10,11]. Detection of lenticonus generally occurs following the onset of renal failure, which usually occurs during the patient’s early middle age. Patients experience a progressive vision loss due to astigmatism caused by the lenticonus; refraction is unable to correct this. Diagnosis is aided by the appearance of a characteristic ‘oil droplet’ in a slit-lamp examination or direct ophthalmoscopy [12]. Typically, over time lenticonus becomes more problematic and leads to deteriorating vision that cannot be corrected with refractive glasses; eventually, the symptoms require treatment, which for most patients involves surgical intervention [13,14]. In slit-lamp biomicroscopy, lenticonus appears as an anterior or posterior protrusion of the lens, extending towards the anterior or vitreous chamber, respectively. Moreover, common retinal observations in Alport syndrome include central, peripheral or perimacular fleck retinopathy, temporal thinning such as loss of the foveal reflex and changes of foveal pigmentation. Other manifestations include lamellar and giant macular hole and the presence of a ‘bull’s eye’ or vitelliform maculopathy [14-18].

Similar to the case presented herein, Al-Mahmood and colleagues reported a case of a young man with Alport syndrome who presented with bilateral anterior lenticonus with progressive posterior lenticonus [5]. However, that patient was younger than the patient described here. Other authors also reported instances of simultaneous bilateral anterior and posterior lenticounus in patients Alport syndrome at presentation [2-4].

In this case, the symptoms exhibited by the patient were consistent with Alport syndrome and associated ocular manifestations. Initially, only anterior lenticonus was present, but subsequently posterior lenticonus developed as a progressive change in the same eye. In identifying anterior lenticonus in an Alport syndrome, the patient ought to alert clinicians to the potential of the presence of a pre-existing posterior lenticonus or to anticipate the potential of it developing over time. The presence of anterior and posterior lenticonus can increase the risks of complications in ophthalmological surgery. Therefore, it is important to be mindful of the thinning and weakening effect that the lens protrusion has on the overlaying capsule. Histopathologic examination of lenticonus shows the anterior lens capsule becomes thinner, and the inner part of the central anterior lens capsule exhibits vertical dehiscences [11, 19]. Therefore, surgeons intending to perform lens extraction surgery and IOL implantation ought to proceed very carefully. Ideally, the patient should be booked for surgery as early as possible. To prevent capsular extension or posterior capsular rupture and lens drop, the surgeon needs to exercise extreme care in the capsulorrhexis step and hydrodissection.

## Conclusions

This is a case report showing that anterior lenticonus, although exceedingly rare to develop in the same patient, have in fact developed in this patient with Alport syndrome with progression to posterior lenticonus. Therefore, surgeons planning to perform lens extraction surgery and intraocular lens implantation must proceed very carefully to avoid potential complications. Also, it is recommended that ophthalmologists be careful in examining patients with Alport syndrome and remain alert to the possibility of the presence of posterior lenticonus or the potential of anterior lenticonus to progress into posterior lenticonus over time. Anterior segment optical coherence tomography (OCT) and Scheimpflug image on Pentacam should be a regular part of patient follow up for early detection.
